# The efficacy and safety of Tanreqing injection combined with western medicine for severe pneumonia: A protocol for systematic review and meta-analysis

**DOI:** 10.1097/MD.0000000000022010

**Published:** 2020-08-28

**Authors:** Lei Wang, Yihua Fan, Jingyu Xu, Huaihan Deng, Chen Geng, Bo Jia

**Affiliations:** a Chengdu University of Traditional Chinese Medicine, Chengdu 610072, Sichuan Province; b First Teaching Hospital of Tianjin University of Traditional Chinese Medicine, Tianjin 300193; c Pengzhou Hospital of Traditional Chinese Medicine, Pengzhou 6119301, Sichuan Province; d Tianjin University of Traditional Chinese Medicine, Tianjin 301617, China.

**Keywords:** meta-analysis, protocol, severe pneumonia, systematic evaluation, Tanreqing injection

## Abstract

**Background:**

Tanreqing injection, as a kind of traditional Chinese medicine injection widely used in clinic, has the effects of clearing heat, reducing phlegm and detoxifying, and avoids the problems of slow effect and complicated decocting of traditional Chinese medicine. Severe pneumonia is a critical disease of the respiratory system, with symptoms such as dyspnea, shortness of breath, high fever, and coma. Clinical studies have found that Tanreqing injection combined with Western medicine has a good effect in the treatment of severe pneumonia. In order to explore the efficacy and safety of Tanreqing injection combined with antibiotics in the treatment of severe pneumonia, we plan to conduct a systematic evaluation and meta-analysis.

**Methods:**

Randomized controlled trials (RCTs) on the treatment of severe pneumonia with Tanreqing injection combined with western medicine were collected by searching PubMed, The Cochrane Library, Embase, Web of Science, CNKI, Wanfang Database, Weipu Database, and China Biomedical Literature Service System (CBM) by computer with the retrieval time from establishment of database to July 2020. Two researchers independently screened and extracted the literature, and finally evaluated the bias risk of the included study, and meta-analysis was conducted using RevMan5.3 software.

**Results:**

The study evaluated the efficacy and safety of Tanreqing injection combined with Western medicine in the treatment of severe pneumonia in terms of total response rate, CURB-65 score, white blood cell count (WBC), antipyretic time (AT), adverse reaction incidence, etc.

**Conclusions:**

This study will provide a reliable evidence-based basis for the clinical application of Tanreqing injection in the treatment of severe pneumonia.

**Ethics and dissemination:**

The private information from individuals will not be published. This systematic review also will not involve endangering participant rights. Ethical approval is not required. The results may be published in a peer-reviewed journal or disseminated in relevant conferences.

**OSF Registration number:**

DOI 10.17605/OSF.IO/SQDMG.

## 1 Introduction

Severe pneumonia is a critical and serious respiratory disease with severe poisoning symptoms or complications,^[1]^ characterized by rapid progress and high fatality rate.^[2]^ Its clinical manifestations mainly include dyspnea, palpitations, hypotension, hyperthermia, shock, disturbance of consciousness, and lung moist rhonchus.^[3]^ Pathogenic microorganisms were detected in 95.20% of patients with severe pneumonia, 49.2% of whom were bacterial infections.^[2]^ Therefore, the use of antibiotics is essential.

Early effective anti-infective treatment is the main method to reduce the mortality of patients with severe pneumonia.^[4]^ But in recent years, with the wide application of antibiotics, the pathogenic bacteria have changed greatly. Bacterial resistance has become a global problem.^[5]^ Meanwhile, as severe pneumonia is often accompanied by serious complications, such as septicemia, empyema, pericarditis, respiratory distress syndrome, multifunctional organ failure, etc,^[6,7]^ the number of deaths due to severe pneumonia is increasing day by day. At present, symptomatic treatment is the main treatment for severe pneumonia, but the effect of conventional treatment is poor.^[8]^ Therefore, combined drug use has gradually become the trend of drug use, and traditional Chinese medicine has its unique advantages in the treatment of severe pneumonia. Tanreqing injection is a new product of Chinese herbal medicine commonly used clinically in recent years, which has the functions of clearing away heat, detoxifying, resolving phlegm, and resisting infection.^[9]^ It is often combined with antibiotics to treat severe pneumonia, with significant curative effect.

As more and more studies on Tanreqing injection in the treatment of severe pneumonia have been conducted in recent years, in order to further understand the significance of its clinical efficacy, this meta-analysis was conducted to provide a more powerful reference for the clinical application of Tanreqing injection in severe pneumonia.

## 2 Methods

### 2.1 Protocol register

This protocol of systematic review and meta-analysis has been drafted under the guidance of the preferred reporting items for systematic reviews and meta-analyses protocols (PRISMA-P). Moreover, it has been registered on open science framework (OSF) on July 26, 2020 (Registration number: DOI 10.17605/OSF.IO/SQDMG).

### 2.2 Ethics

Since this is a protocol with no patient recruitment and personal information collection, the approval of the ethics committee is not required.

### 2.3 Eligibility criteria

#### 2.3.1 Types of studies

Randomized controlled trials (RCTs), regardless of blind method or publication area, whose languages were limited to Chinese and English.

#### 2.3.2 Objects of the study

Patients with severe pneumonia were diagnosed according to *The expert consensus on the Clinical Practice of Severe pneumonia in Emergency Department of China*,^[10]^ with no restriction on gender, age, and source of cases.

#### 2.3.3 Intervention

The control group was treated with western medicine, such as antibiotics, oxygen inhalation, temperature control, electrolyte acid–base balance disorder correction, mechanical ventilation, etc. In the experimental group, Tanreqing injection intravenous dripping was added on the basis of the control group.

#### 2.3.4 Outcome indicator

(1) Primary outcome: the total effective rate. The clinical efficacy evaluation is based on the Guiding Principles for Clinical Research of New Traditional Chinese Medicine Drugs issued by the Ministry of Health of China.^[11]^
(2) Secondary outcomes:
  1. CURB-65 Score, provided on the *Curb-65 Scale for Community-acquired pneumonia*,^[12]^ which included impaired consciousness, blood urea nitrogen, respiratory frequency, blood pressure, and age;
  2. WBC count;
  3. antipyretic time (AT);
  4. Incidence of adverse reactions, adverse reactions include: dizziness, headache, nausea and vomiting, drug skin rash, pruritus, and so on.

### 2.4 Exclusion criteria

(1) As for repeatedly published study, choose the one with the most complete data;
(2) Study whose data is incomplete, and relevant literature cannot be obtained after contacting the author;
(3) Study with the treatment group treated in other traditional Chinese medicine treatments besides Tanreqing injection;
(4) Study with no relevant outcome indicators.

### 2.5 Search strategy

PubMed, The Cochrane Library, Embase, and Web of Science were retrieved with “Tanreqing,” “TRQI,” “Pneumonia,” “Lobar Pneumonia,” etc; Articles related to severe pneumonia were selected after searching; “Tanreqing” and “severe pneumonia” were used as Chinese search terms to search CNKI, Wanfang Database, Weipu database, and China Biomedical Literature Service System (CBM), and use a combination of subject words and free words. In addition, do a manual search in baidu academic and Google academic. The retrieval time was from the establishment of the database to July 2020, and all randomized controlled trials of Tanreqing injection combined with Western medicine in the treatment of severe pneumonia were comprehensively searched. Take Pubmed as an example, and the specific retrieval strategy is shown in Table 1.

**Table 1:**
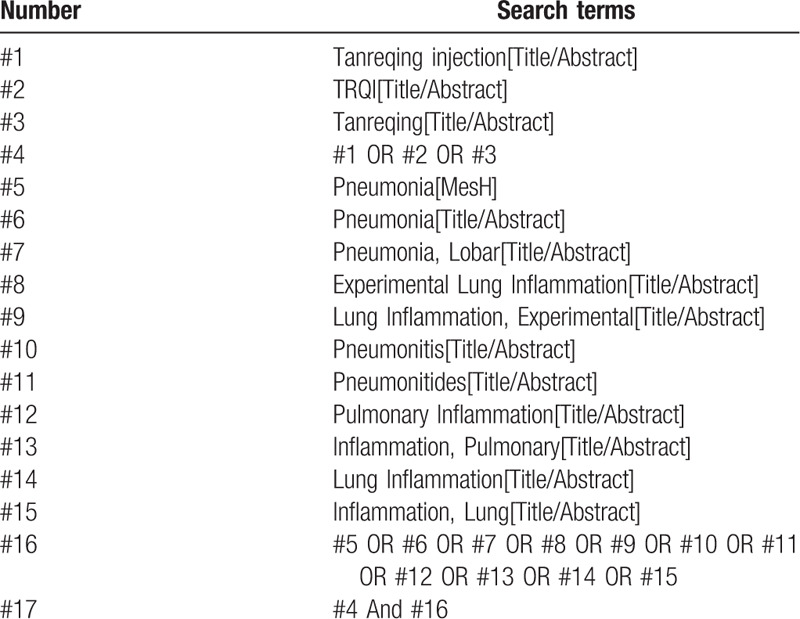
Search strategy in PubMed database.

### 2.6 Data screening and extraction

**Figure 1:**
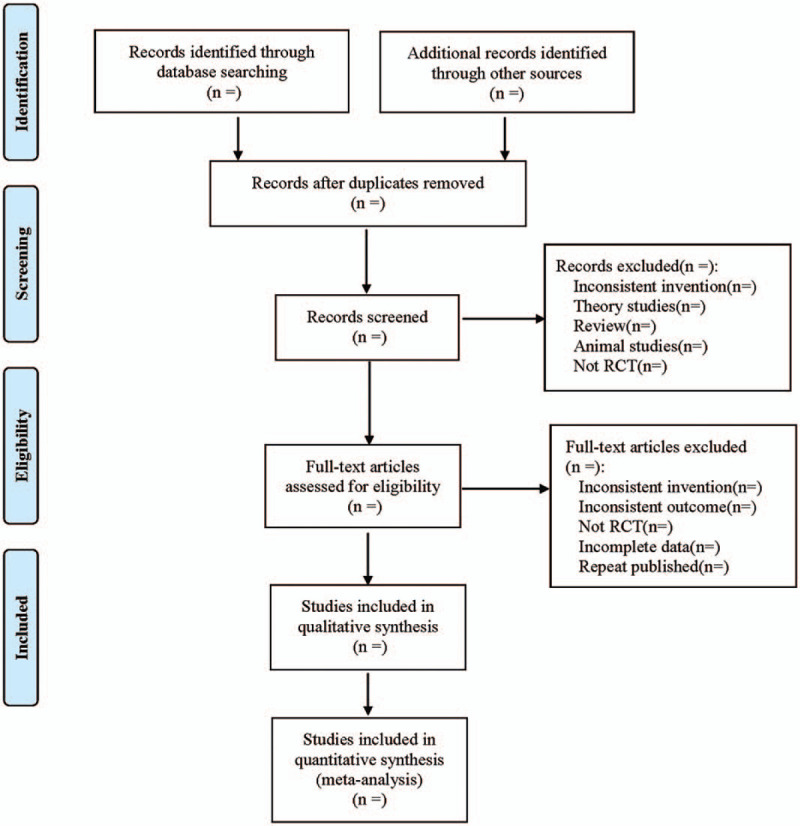
Flow diagram.

Two reviewers conducted literature screening independently. First, EndNote X9 software was used to review the literature in each database, and then the title and abstract of the literature were initially screened. Then, the full text was further screened according to the inclusion and exclusion criteria. If there were different opinions, negotiate with a third party to resolve the differences. If necessary, contact the author of the article by email or telephone for important information about this study. At the same time, Excel 2013 was used to extract relevant information, including:

(1) Clinical research (title, first author, publication date, sample size, sex ratio, average age, average course of disease);
(2) Intervention measures (western medicine treatment measures in control group, such as western medicine name, dose, course of treatment; the western medicine used in the treatment group, as well as the dose, frequency, and course of Tanreqing injection);
(3) Risk bias assessment factors in randomized controlled trials;
(4) Observation index. The literature selection process is shown in Figure 1.

### 2.7 Literature quality evaluation

Using the Cochrane collaboration's tool for assessing risk of bias to assess the risk bias of the included study. According to the performance of the included literature in the above evaluation items, the 2 researchers will give low risk, unclear and high risk judgments one-by-one, and cross-check after completion, respectively. In case of differences, discussion will be conducted. If no agreement can be reached, discussion will be made with the researchers in the third party.

### 2.8 Statistical analysis

#### 2.8.1 Data analysis and processing

The RevMan 5.3 software provided by the Cochrane Collaboration was used for statistical analysis.

(1) For dichotomous variables, relative risk (RR) was used for statistics. For continuous variables, Weighted Mean Difference (WMD) was selected when the tools and units of measurement indicators are the same, Standardized Mean Difference (SMD) was selected with different tools or units of measurement, and all the above were represented by effect value and 95% confidence interval (CI).
(2) Heterogeneity test: *Q* test was used to qualitatively determine inter-study heterogeneity. If *P* ≥ .1, there was no inter-study heterogeneity, If *P* < .1, it indicated inter-study heterogeneity. At the same time, *I*^2^ value was used to quantitatively evaluate the inter-study heterogeneity. If *I*^2^ ≤ 50%, the heterogeneity was considered to be good, and the fixed-effect model was adopted. If *I*^2^ > 50%, it was considered to have significant heterogeneity, the source of heterogeneity would be explored through subgroup analysis or sensitivity analysis. If there was no obvious clinical or methodological heterogeneity, it would be considered as statistical heterogeneity, and the random-effect model would be used for analysis. Descriptive analysis was used if there was significant clinical heterogeneity between the 2 groups and subgroup analysis was not available.

#### 2.8.2 Dealing with missing data

If data is missing or incomplete, we will contact the corresponding author to obtain the missing data. If not, this study will be removed.

#### 2.8.3 Subgroup analysis

To address clinical heterogeneity, we performed a subgroup analysis. According to the age of the patients, the study can be divided into 3 subgroups: minors, young adults, and the elderly. According to the patient's condition, subgroup analysis can be conducted, and according to the Clinical pulmonary infection Score (CPIS score) at admission,^[10]^ it can be divided into 3 subgroups: 0 to 6 points, 7 to 9 points, and 10 to 12 points.

#### 2.8.4 Sensitivity analysis

In order to test the stability of meta-analysis results of outcomes, a one-by-one elimination method will be adopted for sensitivity analysis.

#### 2.8.5 Assessment of reporting biases

For the major outcome indicators, if the included study was ≥10, funnel plot was used to qualitatively detect publication bias. Egger's and Begg's test are used to quantitatively assess potential publication bias.

#### 2.8.6 Evidence quality evaluation

The Grading of Recommendations Assessment, Development, and Evaluation (GRADE)^[13]^ will be used to assess the quality of evidence. It contains 5 domains (bias risk, consistency, directness, precision, and publication bias). And the quality of evidence will be rated as high, moderate, low, and very low.

## 3 Discussion

Severe pneumonia is one of the most common critical diseases in clinical practice. It tends to occur in the elderly and children.^[14–16]^ It is characterized by rapid onset, rapid change of disease condition, and high fatality rate.^[17]^ Severe pneumonia is the leading cause of death among children.^[18]^ Severe pneumonia is common in pathogenic bacteria infection, and pulmonary infection is gradually aggravated due to unreasonable use of antibiotics and patients’ own physical factors, thus causing damage to multiple organs throughout the body^[19]^ and developing into severe pneumonia. This disease belongs to the category of “wind-warm lung-heat disease” (Fengwen Feire Bing), “lung-heat disease” (Feire Bing), “lung carbuncle” (Feiyong), and so on in Chinese medicine. As early as 2000 years ago, there were records of pneumonia-like manifestations in the *Huangdi Neijing • Suwen*. In traditional Chinese medicine, it is believed that the cause of severe pneumonia is deficiency of vital qi or failure to treat by mistake, the inflow of wind-heat pathological qi, and excessive noxious heat is the cause and pathogenesis of severe pneumonia.

Due to the spread of pathogenic bacteria, as well as the impact of environmental pollution and people's living and eating habits, the incidence of pneumonia increases year by year.^[20]^ A large number of clinical practices have shown that the treatment effect of conventional antibiotics for severe pneumonia is increasingly less obvious, while the treatment effect of traditional Chinese medicine combined with antibiotics is significantly better.^[21,22]^ Tanreqing injection has been gradually applied in recent years. It is composed of Scutellaria, bear bile powder, horn powder, honeysuckle, forsythia,^[9]^ among which scutellaria has the functions of clearing heat, drying moisture, antiviral, anti-inflammatory, and lowering blood pressure.^[23]^ Bear bile powder has the functions of reducing phlegm, spasmolysis, detoxifying, inhibiting bacteria and anti-inflammatory, relieving cough, eliminating phlegm, and relieving asthma. Horn powder has significant antipyretic, sedative, and immune effects. Honeysuckle can clear heat and detoxify, and has broad-spectrum antibacterial effect.^[24]^ Forsythia has the ability to ventilate the lung and dissipate phlegm, dispel the wind, and dissipate stagnation.^[23]^ Many studies have shown that Tanreqing injection combined with conventional antibiotics is more effective in treating severe pneumonia. The flavonoids contained in it, mainly baicalin, have strong antibacterial, anti-inflammatory, sedative, and antipyretic effects.^[25,26]^ Experiments have shown that Tanreqing injection has a strong inhibitory effect on streptococcus pneumoniae, B hemolytic streptococcus, *Staphylococcus aureus*, and other pathogenic bacteria.^[27]^ Therefore, in order to systematically evaluate the clinical efficacy of Tanreqing injection in the treatment of severe pneumonia and provide a practical, objective and powerful basis for clinical diagnosis and treatment, we conducted this meta-analysis.

However, this study also has some limitations:

(1) Some of the included studies seldom describe the specific operation of allocation concealment and blind method;
(2) Only English and Chinese literatures were searched due to the limitation on languages. Studies in other languages were ignored, which might lead to certain publication bias;
(3) Due to the critical condition and many complications of severe pneumonia, it is impossible to guarantee that the western medicine treatment measures in the control group are exactly the same, with certain clinical heterogeneity;
(4) Due to the lack of data, this study cannot say whether Tanreqing injection can reduce the death rate of severe pneumonia.

## Author contributions

**Data collection:** Jingyu Xu and Huaihan Deng.

**Literature search:** Lei Wang and Yihua Fan.

**Software operation:** Chen Geng.

**Supervision:** Bo Jia.

**Writing – original draft:** Lei Wang and Yihua Fan.

**Writing – review & editing:** Lei Wang and Bo Jia.
